# Short-Term Prediction of Preeclampsia in Chinese Women Using the Soluble fms-Like Tyrosine Kinase 1/Placental Growth Factor Ratio: A Sub-Analysis of the PROGNOSIS Asia Study

**DOI:** 10.3389/fcvm.2021.602560

**Published:** 2021-08-23

**Authors:** Jinsong Gao, Xianghua Huang, Wen Di, Xiaojing Dong, Wenli Gou, Hong Shi, Zilian Wang, Angela Dietl, Sonja Grill, Martin Hund

**Affiliations:** ^1^Peking Union Medical College Hospital, Chinese Academy of Medical Sciences, Beijing, China; ^2^The Second Hospital of Hebei Medical University, Shijiazhuang, China; ^3^Renji Hospital Affiliated to Shanghai Jiao Tong University School of Medicine, Shanghai, China; ^4^The Second Affiliated Hospital of Chongqing Medical University, Chongqing, China; ^5^The First Affiliated Hospital of Xi'an Jiaotong University, Xi'an, China; ^6^The First Affiliated Hospital of Dalian Medical University, Dalian, China; ^7^The First Affiliated Hospital, Zhongshan (Sun Yat-sen) University, Guangzhou, China; ^8^Roche Diagnostics GmbH, Penzberg, Germany; ^9^Roche Diagnostics International Ltd, Rotkreuz, Switzerland

**Keywords:** soluble fms-like tyrosine kinase 1, placental growth factor, preeclampsia, prediction, China

## Abstract

The diagnosis of preeclampsia in China currently relies on limited clinical signs and unspecific laboratory findings. These are inadequate predictors of preeclampsia development, limiting early diagnosis and appropriate management. Previously, the Prediction of Short-Term Outcome in Pregnant Women with Suspected Preeclampsia Study (PROGNOSIS) and PROGNOSIS Asia demonstrated that a soluble fms-like tyrosine kinase 1 (sFlt-1)/placental growth factor (PlGF) ratio of ≤38 can be used to rule out preeclampsia within 1 week, with negative predictive values of 99.3 and 98.6%, respectively. This is an exploratory sub-analysis of the Chinese cohort (*n* = 225) of the PROGNOSIS Asia study. The primary objectives were to assess the predictive performance of using the sFlt-1/PlGF ratio to rule out preeclampsia within 1 week and to rule in preeclampsia within 4 weeks. The sFlt-1/PlGF ratio was also examined for short-term prediction of fetal adverse outcomes, maternal adverse outcomes, and time to delivery. The overall prevalence of preeclampsia was 17.3%. With the use of an sFlt-1/PlGF ratio of ≤38, the negative predictive value for ruling out preeclampsia within 1 week was 97.3% [95% confidence interval (CI), 93.8–99.1], with a sensitivity of 64.3% and specificity of 85.3%. With the use of an sFlt-1/PlGF ratio of >38, the positive predictive value for ruling in preeclampsia within 4 weeks was 35.0% (95% CI, 20.6–51.7), with a sensitivity of 50.0% and specificity of 86.8%. In the analyses of the sFlt-1/PlGF ratio and fetal adverse outcomes, the area under the receiver operating characteristic curve was 92.8% (95% CI, 83.5–98.7) for ruling out fetal adverse outcomes within 1 week and 79.9% (95% CI, 68.1–90.3) for ruling in fetal adverse outcomes within 4 weeks. An sFlt-1/PlGF ratio of >38 increased the likelihood of imminent delivery 3.3-fold compared with a ratio of ≤38 [hazard ratio, 3.3 (95% CI, 2.1–5.1)]. This sub-analysis confirms the high predictive performance of the sFlt-1/PlGF ratio cutoff of 38 for short-term prediction of preeclampsia in Chinese women, which may help prevent unnecessary hospitalization of women with low risk of developing preeclampsia.

## Introduction

Preeclampsia is a heterogeneous, pregnancy-specific hypertensive disease with multisystem involvement (1). It affects 2–8% of pregnancies worldwide and approximately 1.9% of pregnancies in China (2–4). The relationship between ethnicity and the risk of preeclampsia is well-documented, with some studies reporting the morbidity of the Uygur 2.4 times higher than that of the Han nationality in China (5).

Preeclampsia is a major cause of perinatal and maternal morbidity and mortality (6), with an estimated 2.6 million stillbirths each year, of which 98% occur in low- and middle-income countries. In China, a stillbirth rate of 8.8 per 1,000 births was reported in 2016 (7).

Currently, diagnosis of preeclampsia relies on the presence of new-onset hypertension plus proteinuria, although both are in fact poor predictors of preeclampsia development (8, 9). Diagnosis of preeclampsia in China is largely based on limited clinical information and unspecific laboratory findings, due to a need for timely diagnosis and patient management (10). Consequently, the triage of Chinese women presenting with suspected preeclampsia is challenging (11). This inability to accurately predict preeclampsia may lead to the unnecessary hospitalization of women, or a failure to identify those women who develop preeclampsia, with increased risks for the fetus. A reliable method for the short-term prediction of preeclampsia in Chinese women is therefore needed.

An imbalance of circulating maternal levels of soluble fms-like tyrosine kinase 1 (sFlt-1) and placental growth factor (PlGF) is associated with preeclampsia development (12). The ratio of sFlt-1 to PlGF is elevated in pregnant women 4–5 weeks prior to and during the clinical onset of preeclampsia (13). The sFlt-1/PlGF ratio has also been shown to discriminate between different types of pregnancy-related hypertensive disorders when combined with other clinical biomarkers (14, 15). The Prediction of Short-Term Outcome in Pregnant Women with Suspected Preeclampsia Study (PROGNOSIS) and PROGNOSIS Asia studies have previously shown that an sFlt-1/PlGF ratio of ≤38 can rule out preeclampsia within 1 week, while a ratio of >38 can rule in preeclampsia within 4 weeks (16–18).

This exploratory sub-analysis of the PROGNOSIS Asia study was performed to assess the performance of the sFlt-1/PlGF ratio for short-term prediction of preeclampsia and pregnancy-related adverse events in Chinese women.

## Materials and Methods

### Study Overview

PROGNOSIS Asia was a prospective, blinded, non-interventional, multicenter study that enrolled 764 women with suspected preeclampsia at 25 sites across Asia (China, Hong Kong, Japan, Singapore, South Korea, and Thailand) between December 2014 and December 2016; results of the primary analysis have been reported previously (18), as have results of an exploratory sub-analysis of the Japanese cohort (19). The Chinese cohort was enrolled at seven sites in mainland China between March 2015 and September 2016.

Local ethics committees and institutional review boards at each site approved the protocol prior to study initiation, and approval from the Human Genetic Resources Administration of China was obtained (May 2016). The study was performed in compliance with the International Conference on Harmonization guidelines for Good Clinical Practice and the principles of the Declaration of Helsinki, and all participants provided written informed consent.

### Study Participants

Eligible participants included pregnant women ≥18 years of age, at gestational age 20 weeks + 0 days to 36 weeks + 6 days, with clinical suspicion of preeclampsia according to protocol-defined criteria, previously published by Bian et al. (18). Women who had manifest preeclampsia or a confirmed diagnosis of hemolysis, elevated liver enzymes, low platelet count (HELLP) syndrome; those with multiple pregnancies or a confirmed diagnosis of a fetal chromosomal abnormality; and those who had received treatment with an investigational medicine within 90 days before enrollment were excluded. Diagnostic criteria for preeclampsia and preeclampsia-related disorders were based on the International Society for the Study of Hypertension in Pregnancy guidelines (ISSHP) (9), as described in Zeisler et al. (16).

### Study Procedures

Demographic information and medical history for each participant were collected at screening. Assessments were made at all study visits: visit 1 (baseline), visit 2 (7–14 days from baseline), visit 3 (24–32 days from baseline), at delivery, and postpartum. In addition, unscheduled visits occurred in the event of pregnancy complications. Clinical data were collected at all study visits and recorded in an electronic case report form, with regular monitoring.

Maternal serum samples (2 ml) were collected at visits 1–3 according to standard operating procedures and were stored frozen until analysis. Samples were analyzed by an independent accredited laboratory (Covance Central Laboratory Service, Shanghai, China). Maternal serum concentrations of sFlt-1 and PlGF (both measured in picograms per milliliter) were determined using the fully automated Elecsys® sFlt-1 and PlGF immunoassays on cobas e analyzers (Roche Diagnostics, Mannheim, Germany) (20). To prevent results from influencing clinical decision making, sample measurements were performed after study completion.

### Analysis Objectives/Endpoints

The primary objectives were to assess the performance of an Elecsys sFlt-1/PlGF ratio cutoff of ≤38 to predict the absence of preeclampsia within 1 week and a cutoff of >38 to predict the occurrence of preeclampsia within 4 weeks. Based on the results of PROGNOSIS and PROGNOSIS Asia, selected secondary objectives were investigated in this Chinese cohort in an exploratory manner. These secondary objectives included investigation of the sFlt-1/PlGF ratio for short-term prediction of fetal and maternal adverse outcomes (FAOs and MAOs), and assessment of the correlation between the sFlt-1/PlGF ratio and time to delivery. FAOs examined included perinatal/fetal death, delivery <34 weeks, fetal growth restriction, placental abruption, neonatal respiratory distress syndrome, necrotizing enterocolitis, intraventricular hemorrhage, hospitalization separate from the mother, admission to neonatal intensive care unit, hypoxia, neurologic injury, and asphyxia. MAOs were defined as any preeclampsia-related adverse outcome other than preeclampsia/eclampsia/HELLP syndrome, including maternal death, pulmonary edema, acute renal failure, and cerebral hemorrhage.

### Statistical Analyses

A sample determination formula according to Pepe (21) was applied. Sample size calculations were based on the Schatzkin criterion and were performed for the entire study population of PROGNOSIS Asia (not for analyses in subsets). Data analysis followed a statistical analysis plan and used SAS 9.4 software (SAS, Cary, NC, USA), and R 3.2.2 and R 3.4.0 software (R Foundation, Vienna, Austria). All data were transferred to the sponsor for merging and analysis at the end of the study. The sFlt-1/PlGF ratio was calculated by the Roche biostatistics department (Penzberg, Germany). Descriptive statistics were reported as median and interquartile range (IQR) for continuous data, and as absolute and relative frequencies for count data. The predictive performance of the sFlt-1/PlGF ratio was assessed by estimating negative predictive value (NPV), positive predictive value (PPV), sensitivity, specificity, and area under the receiver operating characteristic (ROC) curve, each derived with a corresponding 95% confidence interval (CI). The impact of the sFlt-1/PlGF ratio on the remaining pregnancy duration at the time of blood sampling was estimated by Cox regression, dichotomized by sFlt-1/PlGF ratio status (≤38 vs. >38), and adjusted for gestational age and final preeclampsia status.

## Results

### Analysis Population

Of the 250 women enrolled in the Chinese cohort, 225 (90%) were evaluable and included in the current analysis (Supplementary Figure 1). Median age at baseline and gestational age at baseline were comparable between women who developed preeclampsia and those who did not develop preeclampsia (*p* = 0.488 and *p* = 0.327, respectively; Table 1). Median body mass index and blood pressure were higher in women who developed preeclampsia than in those who did not develop preeclampsia (*p* < 0.001 and *p* < 0.001, respectively), whereas median gestational age at delivery and median height and weight of the neonate were lower in women who developed preeclampsia than in those who did not develop preeclampsia (*p* < 0.001, *p* < 0.001, and *p* < 0.001, respectively). The overall prevalence of preeclampsia was 17.3%, with 6.2% of women diagnosed within 1 week and 12.4% diagnosed within 4 weeks. The most common reasons for suspected preeclampsia were as follows: “new onset of hypertension” (54.7%), “new onset of protein in urine” (23.6%), and “abnormal uterine perfusion” (23.6%).

**Table 1:** Participant demographics and characteristics at baseline for the whole cohort and according to preeclampsia status.

**Characteristic** ^**a**^ ^,^ ^**b**^	**All women (*N* = 225)**	**No preeclampsia at any time (*n* = 186)**	**Preeclampsia at any time (*n* = 39)**
Age, years	31.0 (28.0–34.0)	31.0 (29.0–34.0)	31.0 (28.0–34.0)
Gestational age of pregnancy at baseline, weeks	30.6 (26.7–33.7)	30.5 (26.7–34.0)	30.9 (26.3–32.3)
Gestational age of pregnancy at delivery, weeks	38.1 (36.7–39.1)	38.4 (37.1–39.3)	36.3 (34.3–37.4)
Blood pressure at baseline, mmHg			
Systolic	138.0 (125.0–148.0)	136.5 (121.0–146.0)	146.0 (135.0–160.0)
Diastolic	91.0 (81.0–98.0)	90.0 (78.0–97.0)	94.0 (90.0–107.0)
Pre-pregnancy BMI, kg/m^2^	23.8 (20.8–26.5)	23.4 (20.7–25.8)	26.2 (23.3–30.5)
Reasons for suspected preeclampsia, *n* (%)^c^			
New onset of hypertension	123 (54.7)	102 (54.8)	21 (53.8)
Aggravation of preexisting hypertension	30 (13.3)	21 (11.3)	9 (23.1)
New onset of protein in urine	53 (23.6)	38 (20.4)	15 (38.5)
Aggravation of preexisting proteinuria	3 (1.3)	1 (0.5)	2 (5.1)
Epigastric pain	0	0	0
Visual disturbances	0	0	0
Abnormal uterine perfusion	53 (23.6)	45 (24.2)	8 (20.5)
Partial HELLP syndrome	7 (3.1)	6 (3.2)	1 (2.6)
Height of neonate, cm	49 (47–50)	50 (48–50)	47 (44–50)
Weight of neonate, g	2,950 (2,440–3,350)	2,980 (2,600–3,390)	2,310 (1,800–3,080)

*BMI, body mass index; HELLP, hemolysis, elevated liver enzymes, low platelet count*.

a *Data are reported as median (interquartile range), unless stated otherwise*.

b *p-values were calculated using the Mann–Whitney U-test for continuous variables and Fisher's exact test for categorical variables. Statistically significant differences were observed between women who did not develop preeclampsia at any time and women who developed preeclampsia for the following characteristics: gestational age at delivery (p < 0.001), systolic blood pressure at baseline (p < 0.001), diastolic blood pressure at baseline (p < 0.001), maximum systolic blood pressure (p < 0.001), maximum diastolic blood pressure (p < 0.001), pre-pregnancy BMI (p < 0.001), height of neonate (p < 0.001), weight of neonate (p < 0.001), and new onset of protein in urine (p = 0.022). For the remaining characteristics, p-values were as follows between the two groups: age (p = 0.488), gestational age at visit 1 (p = 0.327), new onset of hypertension (p = 1), aggravation of preexisting hypertension (p = 0.067), aggravation of preexisting proteinuria (p = 0.078), suspected intrauterine growth restriction or abnormal uterine perfusion (p = 0.684), and partial HELLP syndrome (p = 1)*.

c *Some women had suspected preeclampsia for more than one reason*.

### Short-Term Prediction of Preeclampsia

**Figure 1:**
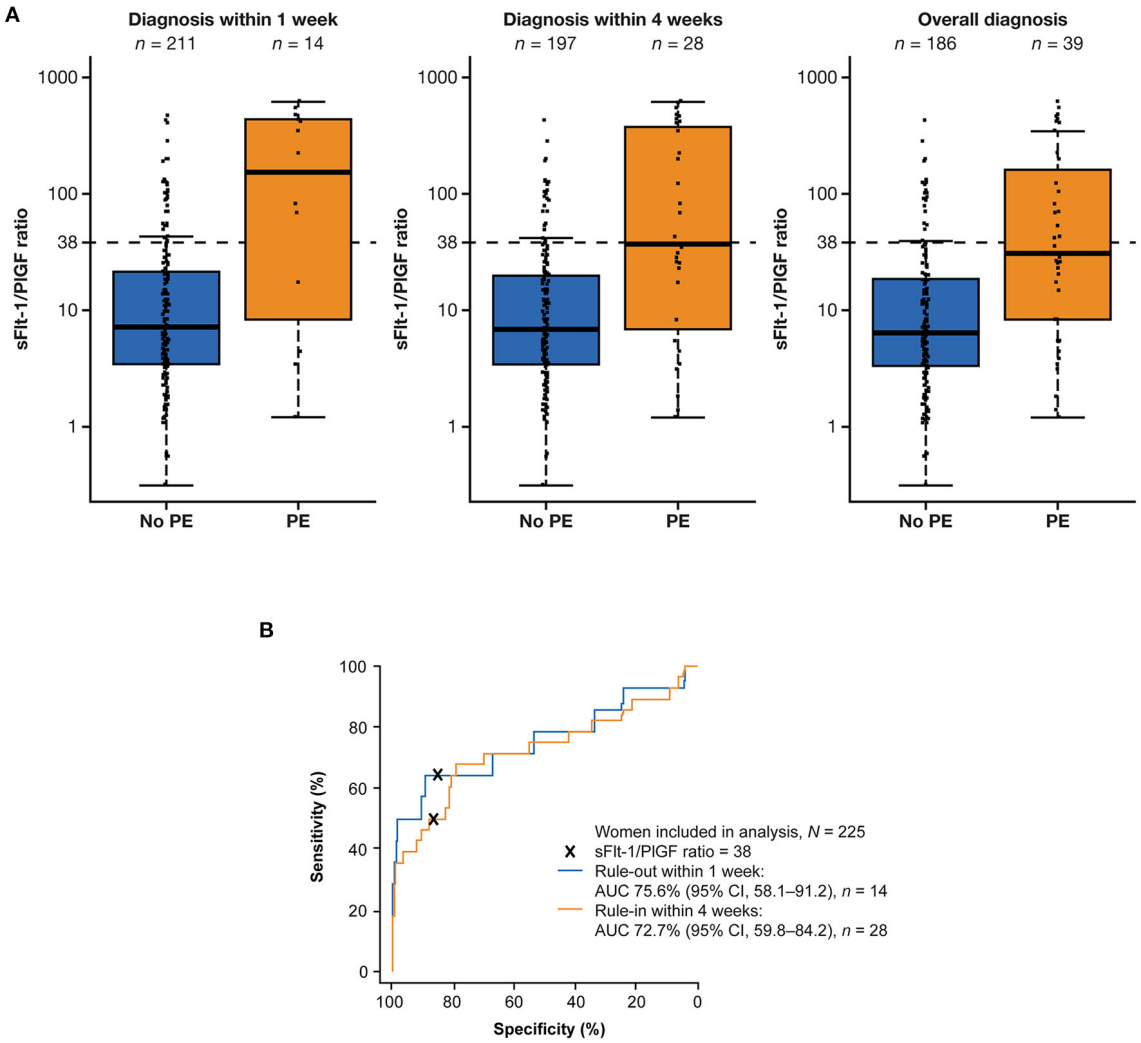
Performance of the sFlt-1/PlGF ratio for predicting preeclampsia within 1 and 4 weeks. **(A)** Distribution of sFlt-1/PlGF ratios at baseline for participants who developed or did not develop preeclampsia within 1 week, within 4 weeks, and overall.^a^ **(B)** Performance of the ratio for ruling out preeclampsia within 1 week (blue) and ruling in preeclampsia within 4 weeks (orange). ^a^The boxes represent the first and third quartiles and the median value. The whiskers represent values that are 1.5 × the IQR or shorter in cases where the minimal/maximal value lies within 1.5 × the IQR in log scale. AUC, area under the curve; CI, confidence interval; IQR, interquartile range; PE, preeclampsia; PlGF, placental growth factor; sFlt-1, soluble fms-like tyrosine kinase 1.

Median sFlt-1/PlGF ratios were elevated among women who developed preeclampsia within 1 week compared with women who did not develop preeclampsia within 1 week (151.68 vs. 7.18). The same trend was observed after 4 weeks (36.97 vs. 6.84) and overall (31.21 vs. 6.43) (Figure 1A). The sFlt-1/PlGF ratio showed moderate sensitivity and high specificity for ruling out preeclampsia within 1 week (ratio ≤38) and ruling in preeclampsia within 4 weeks (ratio >38) (Table 2). Based on an sFlt-1/PlGF ratio of ≤38, the NPV for ruling out preeclampsia within 1 week was 97.3% (95% CI, 93.8–99.1). The area under the ROC curve for the sFlt-1/PlGF ratio ruling out preeclampsia within 1 week was 75.6% (95% CI, 58.1–91.2) (Figure 1B). With the use of an sFlt-1/PlGF ratio of >38, the PPV for ruling in preeclampsia within 4 weeks was 35.0% (95% CI, 20.6–51.7). The area under the ROC curve for the sFlt-1/PlGF ratio ruling in preeclampsia within 4 weeks was 72.7% (95% CI, 59.8–84.2) (Figure 1B).

**Table 2:** Performance of the sFlt-1/PlGF ratio using a cutoff of 38 for short-term prediction of preeclampsia.

**Preeclampsia**	**NPV, % (95% CI)**	**PPV, % (95% CI)**	**Sensitivity, % (95% CI)**	**Specificity, % (95% CI)**
Within 1 week	**97.3 (93.8–99.1)** ^a^	22.5 (10.8–38.5)	64.3 (35.1–87.2)	85.3 (79.8–89.8)
Within 4 weeks	92.4 (87.6–95.8)	**35.0 (20.6–51.7)** ^a^	50.0 (30.6–69.4)	86.8 (81.3–91.2)

*CI, confidence interval; NPV, negative predictive value; PlGF, placental growth factor; PPV, positive predictive value; sFlt-1, soluble fms-like tyrosine kinase 1*.

a *Primary study objectives; for rule out of preeclampsia within 1 week, an sFlt-1/PlGF ratio of ≤38 was used. For rule in of preeclampsia within 4 weeks, an sFlt-1/PlGF ratio of >38 was used*.

### Short-Term Prediction of Pregnancy-Related Adverse Outcomes

**Figure 2:**
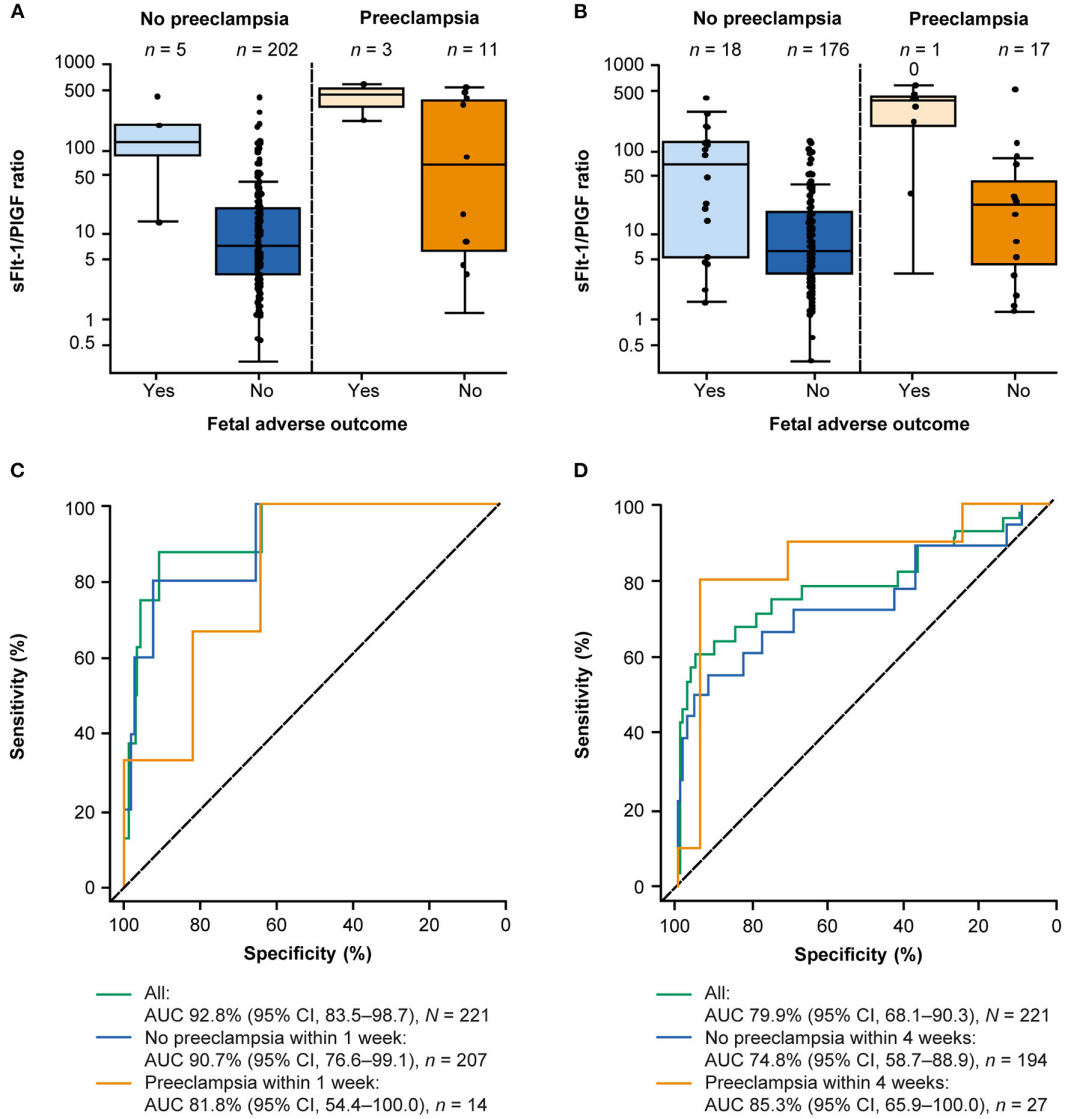
Performance of the sFlt-1/PlGF ratio for predicting FAOs within 1 and 4 weeks.^a^ **(A)** Distribution of the sFlt-1/PlGF ratio at baseline according to FAO status and preeclampsia status within 1 week and **(B)** within 4 weeks.^b^ **(C)** Performance of the sFlt-1/PlGF ratio for short-term prediction of FAOs within 1 week and **(D)** within 4 weeks. ^a^A total of 221 participants from China were eligible for this analysis. ^b^The boxes represent the first and third quartiles and the median value. The whiskers represent values that are 1.5 × the IQR or shorter in cases where the minimal/maximal value lies within 1.5 × the IQR in log scale. AUC, area under the curve; CI, confidence interval; FAO, fetal adverse outcome; IQR, interquartile range; PlGF, placental growth factor; sFlt-1, soluble fms-like tyrosine kinase 1.

Two hundred twenty-one participants were eligible for analysis of the sFlt-1/PlGF ratio and prediction of FAOs. FAOs occurred in eight women within 1 week and 28 women within 4 weeks. The sFlt-1/PlGF ratio was higher in women with ≥1 FAO than in women with no FAOs (Table 3), with the highest sFlt-1/PlGF ratios observed in women who both were diagnosed with preeclampsia and experienced an FAO; this trend was consistent at 1 and 4 weeks (Figures 2A,B). In the ROC curve analysis, the area under the curve was 92.8% (95% CI, 83.5–98.7) for ruling out FAOs within 1 week and 79.9% (95% CI, 68.1–90.3) for ruling in FAOs within 4 weeks (Figures 2C,D). None of the participants included in the Chinese cohort experienced an MAO (as defined by the study protocol), which did not allow for analysis of the sFlt-1/PlGF ratio to predict these outcomes.

**Table 3:** Distribution of sFlt-1/PlGF ratios by FAO status within 1 and 4 weeks in all women and by preeclampsia status.^a^

**FAO status (without/with preeclampsia** ^**b**^ **)**	***N***	**sFlt-1/PlGF ratio, median (IQR)**
FAO within 1 week	8	208.6 (105.8–431.3)
Women without preeclampsia within 1 week	5	124.8 (86.9–195.9)
Women with preeclampsia within 1 week	3	435.3 (221.3–611.5)
No FAO within 1 week	213	7.2 (3.4–21.8)
Women without preeclampsia within 1 week	202	7.1 (3.4–20.8)
Women with preeclampsia within 1 week	11	67.7 (4.4–417.4)
FAO within 4 weeks	28	119.4 (17.0–310.7)
Women without preeclampsia within 4 weeks	18	67.9 (5.2–124.8)
Women with preeclampsia within 4 weeks	10	376.2 (198.1–435.3)
No FAO within 4 weeks	193	6.8 (3.4–19.7)
Women without preeclampsia within 4 weeks	176	6.5 (3.4–18.2)
Women with preeclampsia within 4 weeks	17	22.7 (4.4–42.7)

*FAO, fetal adverse outcome; HELLP, hemolysis, elevated liver enzymes, low platelet count; IQR, interquartile range; PlGF, placental growth factor; sFlt-1, soluble fms-like tyrosine kinase 1*.

a *221 participants from China were eligible for this analysis*.

b *Preeclampsia/eclampsia/HELLP syndrome*.

### Correlation Between the Soluble fms-Like Tyrosine Kinase 1/Placental Growth Factor Ratio and Time to Delivery

**Figure 3:**
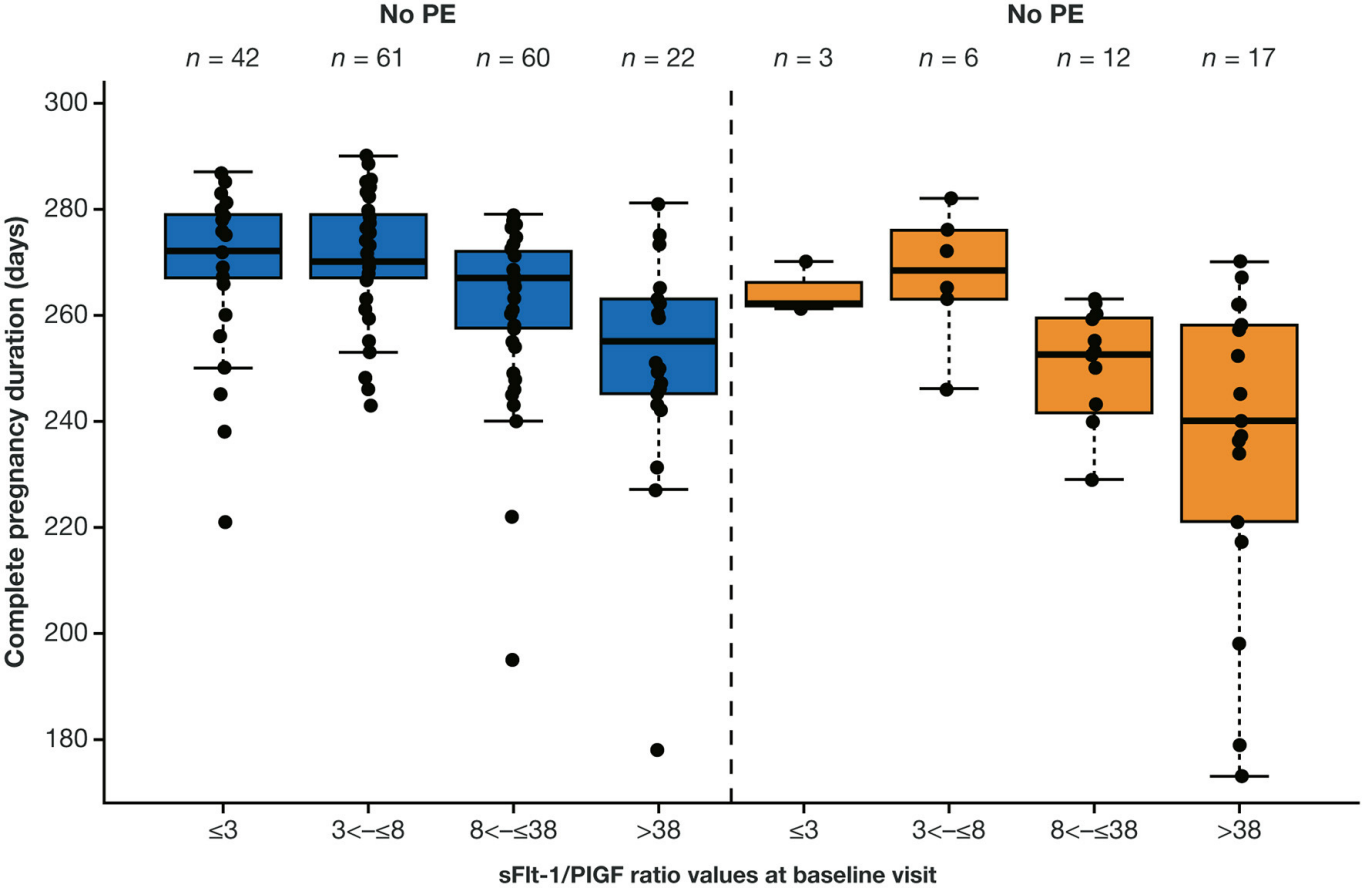
Distribution of complete pregnancy duration according to sFlt-1/PlGF ratio at baseline visit by overall preeclampsia status.^a,b^ ^a^A total of 223 participants from China were eligible for this analysis. ^b^The boxes represent the first and third quartiles and the median value. The whiskers represent values that are IQR or shorter in cases where the minimal/maximal value lies within 1.5 × the IQR. The boxes represent the median and IQR in log scale. IQR, interquartile range; PE, preeclampsia; PlGF, placental growth factor; sFlt-1, soluble fms-like tyrosine kinase 1.

Data for analysis of sFlt-1/PlGF ratio and time to delivery were available for 223 eligible participants. Higher sFlt-1/PlGF ratios were associated with increasingly shorter pregnancy duration leading to preterm delivery (Figure 3), both for women who developed preeclampsia and for those who did not. With the use of Cox regression analysis, the likelihood of imminent delivery (day of test) was 3.3-fold (95% CI, 2.1–5.1) higher in women with an sFlt-1/PlGF ratio >38 vs. ≤38, irrespective of preeclampsia status.

## Discussion

The present sub-analysis validates the predictive value of the sFlt-1/PlGF ratio cutoff of 38 for short-term prediction of preeclampsia, FAOs, and pre-term delivery in Chinese women with clinically suspected preeclampsia. The high NPV [97.3% (95% CI, 93.8–99.1)] of the sFlt-1/PlGF ratio (cutoff ≤38) observed in this sub-analysis allows clinicians to rule out preeclampsia within 1 week in Chinese women with a high degree of confidence, thus supporting clinical decision making on whether to hospitalize patients or not. This has the potential to reduce unnecessary hospitalizations and interventions for women at low risk of preeclampsia, who may instead be monitored and managed in an outpatient setting. The sFlt-1/PlGF ratio >38 provides a PPV of 35.0% (95% CI, 20.6–51.7) for ruling in preeclampsia within 4 weeks in Chinese women. This is higher than that of other predictors such as high blood pressure, which have been shown to have a PPV of 20% for detecting preeclampsia (22).

For the first time, PROGNOSIS Asia demonstrated the value of determining the sFlt-1/PlGF ratio for the short-term prediction of preeclampsia and pregnancy-related adverse outcomes in Asian women with signs and symptoms of preeclampsia (18). The Chinese cohort showed findings consistent with the overall PROGNOSIS Asia study population (18). Baseline characteristics were comparable between the Chinese cohort and the overall population; however, prevalence of preeclampsia was higher in the Chinese cohort (17.3%) compared with the overall population (14.4%). The NPVs for ruling out preeclampsia within 1 week in the Chinese cohort and overall population were 97.3 and 98.6%, respectively. While the PPV for ruling in preeclampsia within 4 weeks was higher in the Chinese cohort (35.0%) vs. the overall population (30.3%), this may be explained by a higher prevalence of preeclampsia in the Chinese cohort compared with overall prevalence (17.3 vs. 14.4%, respectively). The area under the ROC curve for the Chinese cohort and overall population was 92.8 and 91.0%, respectively, for ruling out any FAO within 1 week, and 79.9 and 83.1%, respectively, for ruling in any FAO within 4 weeks. The risk of imminent delivery in women with an sFlt-1/PlGF ratio >38 vs. ≤38 was also comparable between cohorts, with a 3.3-fold and 3.5-fold higher risk observed in the Chinese cohort and overall population, respectively. For all the above results, the 95% CIs in the Chinese cohort overlapped with those from the overall population.

Findings from the Chinese cohort were also consistent with those of an exploratory sub-analysis of the Japanese cohort from PROGNOSIS Asia (19). In the Japanese cohort, overall prevalence of preeclampsia was lower compared with the Chinese cohort (13.3 vs. 17.3%). Median blood pressure at baseline for women who developed preeclampsia at any time was also lower in the Japanese cohort (systolic, 138 vs. 146 mmHg; diastolic, 84 vs. 94 mmHg), while median age was higher in the Japanese cohort compared with the Chinese cohort (35.0 vs. 31.0 years).

The NPV for ruling out preeclampsia within 1 week in the Chinese cohort was lower than that of the Japanese cohort (97.3 vs. 100%), while the PPV for ruling in preeclampsia within 4 weeks was higher in the Chinese cohort compared with the Japanese cohort (35.0 vs. 32.4%). As previously stated, this may be attributed to the higher prevalence of preeclampsia in the Chinese cohort compared with the Japanese cohort (17.3 vs. 13.3%). The area under the ROC curve for ruling out any FAO within 1 week was lower in the Chinese cohort compared with the Japanese cohort (92.8 vs. 93.0%), while risk of imminent delivery in women with an sFlt-1/PlGF ratio >38 vs. ≤38 was higher in the Chinese cohort (3.3-fold vs. 2.8-fold). Again, for all of the above results, 95% CIs in the Chinese cohort overlapped with those from the Japanese cohort.

Our findings were also in line with those of the PROGNOSIS study, in which a 17.8% incidence rate of preeclampsia and/or HELLP syndrome was reported in the validation cohort (74.5% Caucasian) (16). Baseline characteristics were comparable between the Chinese cohort and the PROGNOSIS validation cohort. Moreover, predictive performance of the sFlt-1/PlGF ratio was similar between the PROGNOSIS validation cohort and the Chinese cohort for NPV for ruling out preeclampsia within 1 week (99.3 vs. 97.3%), PPV for ruling in preeclampsia within 4 weeks (36.7 vs. 35.0%), and area under the ROC curve (1 week, 86.1 vs. 75.6%; 4 weeks, 82.3 vs. 72.7%).

The present findings are also consistent with data from the randomized Interventional Study Evaluating the Short-Term Prediction of Preeclampsia/Eclampsia In Pregnant Women With Suspected Preeclampsia (INSPIRE) study, which examined the clinical utility of the sFlt-1/PlGF ratio using a cutoff of 38 (23). The NPV for ruling out preeclampsia within 1 week in the present analysis was 97.3% compared with 100% in INSPIRE (with standard clinical management plus sFlt-1/PlGF ratio; 99.2% using the ratio only).

The findings of this analysis are applicable to Chinese women with clinically suspected preeclampsia and are supported by previous studies demonstrating the predictive value of the sFlt-1/PlGF ratio using a cutoff of 38 in Asian and Caucasian women (16, 18). The potential value of the sFlt-1/PlGF ratio may also extend beyond the prediction of preeclampsia, with other investigators reporting the utility of the ratio for aiding in the diagnosis of early-onset preeclampsia in Chinese women (5, 10).

A strength of this sub-analysis was the use of a well-defined cohort recruited across multiple centers in China. Furthermore, diagnostic criteria were based on ISSHP criteria, ensuring comparability of study results with the PROGNOSIS and PROGNOSIS Asia studies (16). However, our analysis has some limitations. As an exploratory sub-analysis of the PROGNOSIS Asia study, the analysis was not powered for the Chinese cohort presented but rather for the primary analysis of PROGNOSIS Asia. Herein, we used the Elecsys sFlt-1 and PlGF immunoassays to apply the sFlt-1/PlGF ratio cutoff of 38 to predict preeclampsia, but the use of assays from other manufacturers may require a different optimal cutoff. For example, it has been reported that cutoffs for the Elecsys sFlt-1/PlGF ratio are not transferrable to the Brahms Kryptor sFlt-1/PlGF immunoassay (24, 25). In addition, PROGNOSIS Asia was an observational, rather than interventional, study. Further studies should evaluate the real-world utility of the sFlt-1/PlGF ratio for predicting preeclampsia in China.

## Conclusions

This sub-analysis of the Chinese cohort of the PROGNOSIS Asia study confirms the high predictive performance of the Elecsys sFlt-1/PlGF ratio cutoff of 38 for short-term prediction of preeclampsia in Chinese women. The high NPV of 97.3% may enable physicians to rule out preeclampsia within 1 week, which may help to prevent unnecessary hospitalization of women with suspected preeclampsia.

## Data Availability Statement

The data that support the findings of this study are available from Roche Diagnostics International Ltd, but restrictions apply to the availability of these data, which were used under license for the current study, and so are not publicly available. Data are, however, available from the authors upon reasonable request and with permission of Roche Diagnostics International Ltd.

## Author Contributions

JG, XH, WD, XD, WG, HS, and ZW were involved in collection and interpretation of data. AD, SG, and MH were involved in study design and concept development, analysis, and interpretation of data. All authors contributed to the article and approved the submitted version.

## Conflict of Interest

AD and SG are employees of Roche Diagnostics GmbH. MH is an employee of Roche Diagnostics International Ltd and holds stock in F. Hoffmann-La Roche. MH also reports being an inventor of patents related to sFlt-1/PlGF or endoglin/PlGF ratio to rule out onset of preeclampsia in pregnant women within a certain time period PCT/EP2013/063115 and the dynamic of sFlt-1 or endoglin/PlGF ratio as indicator for imminent preeclampsia and HELLP syndrome PCT/EP2012/072157. The remaining authors declare that the research was conducted in the absence of any commercial or financial relationships that could be construed as a potential conflict of interest.

## Publisher's Note

All claims expressed in this article are solely those of the authors and do not necessarily represent those of their affiliated organizations, or those of the publisher, the editors and the reviewers. Any product that may be evaluated in this article, or claim that may be made by its manufacturer, is not guaranteed or endorsed by the publisher.

## References

[1] Chaiworapongsa T Chaemsaithong P Yeo L Romero R. Pre-eclampsia part 1: current understanding of its pathophysiology. Nat Rev Nephrol. (2014) 10:466–80. 10.1038/nrneph.2014.102 25003615 PMC5893150

[2] Ananth CV Keyes KM Wapner RJ. Pre-eclampsia rates in the United States, 1980-2010: age-period-cohort analysis. BMJ. (2013) 347:f6564. 10.1136/bmj.f6564 24201165 PMC3898425

[3] World Health Organisation. The World Health Report 2005 – Make Every Mother and Child Count. (2005). Available online at: http://www.who.int/whr/2005/en/ (accessed May 29, 2020).

[4] Xiao J Shen F Xue Q Chen G Zeng K Stone P. Is ethnicity a risk factor for developing preeclampsia? An analysis of the prevalence of preeclampsia in China. J Hum Hypertens. (2014) 28:694–8. 10.1038/jhh.2013.148 24430700

[5] Ding G Liping L Moli D Wuliyeti A Shaohe Z Huijuan W. A study of the association between the sFlt-1/PIGF ratio and preeclampsia in Xinjiang Uygur autonomous region of China. Artif Cells Nanomed Biotechnol. (2018) 46(Suppl. 3):S281–6. 10.1080/21691401.2018.1491480 30831776

[6] Duley L. The global impact of pre-eclampsia and eclampsia. Semin Perinatol. (2009) 33:130–7. 10.1053/j.semperi.2009.02.010 19464502

[7] Zhu J Liang J Mu Y Li X Guo S Scherpbier R. Sociodemographic and obstetric characteristics of stillbirths in China: a census of nearly 4 million health facility births between 2012 and 2014. Lancet Global health. (2016) 4:e109–18. 10.1016/S2214-109X(15)00271-5 26795603

[8] Seely EW Solomon CG. Improving the prediction of preeclampsia. N Engl J Med. (2016) 374:83–4. 10.1056/NEJMe1515223 26735997

[9] Tranquilli AL Dekker G Magee L Roberts J Sibai BM Steyn W. The classification, diagnosis and management of the hypertensive disorders of pregnancy: A revised statement from the ISSHP. Pregn Hypertens. (2014) 4:97–104. 10.1016/j.preghy.2014.02.001 26104417

[10] Lou WZ Jiang F Hu J Chen XX Song YN Zhou XY. Maternal serum angiogenic factor sFlt-1 to PlGF ratio in preeclampsia: a useful marker for differential diagnosis and prognosis evaluation in Chinese women. Dis Markers. (2019) 2019:6270187. 10.1155/2019/6270187 31396294 PMC6664509

[11] Duley L Meher S Abalos E. Management of pre-eclampsia. BMJ. (2006) 332:463–8. 10.1136/bmj.332.7539.463 16497761 PMC1382544

[12] Vatten LJ Eskild A Nilsen TI Jeansson S Jenum PA Staff AC. Changes in circulating level of angiogenic factors from the first to second trimester as predictors of preeclampsia. Am J Obstetr Gynecol. (2007) 196:239.e1–6. 10.1016/j.ajog.2006.10.909 17346536

[13] Levine RJ Lam C Qian C Yu KF Maynard SE Sachs BP. Soluble endoglin and other circulating antiangiogenic factors in preeclampsia. N Engl J Med. (2006) 355:992–1005. 10.1056/NEJMoa055352 16957146

[14] Verlohren S Herraiz I Lapaire O Schlembach D Moertl M Zeisler H. The sFlt-1/PlGF ratio in different types of hypertensive pregnancy disorders and its prognostic potential in preeclamptic patients. Am J Obstetr Gynecol. (2012) 206:58.e1–8. 10.1016/j.ajog.2011.07.037 22000672

[15] Stepan H Hund M Andraczek T. Combining biomarkers to predict pregnancy complications and redefine preeclampsia: the angiogenic-placental syndrome. Hypertension. (2020) 75:918–26. 10.1161/HYPERTENSIONAHA.119.13763 32063058 PMC7098437

[16] Zeisler H Llurba E Chantraine F Vatish M Staff AC Sennstrom M. Predictive value of the sFlt-1:PlGF ratio in women with suspected preeclampsia. N Engl J Med. (2016) 374:13–22. 10.1056/NEJMoa1414838 26735990

[17] Zeisler H Llurba E Chantraine FJ Vatish M Staff AC Sennstrom M. Soluble fms-like tyrosine kinase-1 to placental growth factor ratio: ruling out pre-eclampsia for up to 4 weeks and value of retesting. Ultrasound Obstetr Gynecol. (2019) 53:367–75. 10.1002/uog.19178 30014562 PMC6590225

[18] Bian X Biswas A Huang X Lee KJ Li TK Masuyama H. Short-term prediction of adverse outcomes using the sFlt-1 (soluble fms-like tyrosine kinase 1)/PLGF (placental growth factor) ratio in Asian women with suspected preeclampsia. Hypertension. (2019) 74:164–72. 10.1161/HYPERTENSIONAHA.119.12760 31188674 PMC6587370

[19] Ohkuchi A Saito S Yamamoto T Minakami H Masuyama H Kumasawa K. Short-term prediction of preeclampsia using the sFlt-1/PlGF ratio: a subanalysis of pregnant Japanese women from the PROGNOSIS Asia study. Hypertens Res. (2021) 44:813–21. 10.1038/s41440-021-00629-x 33727707 PMC8255209

[20] Verlohren S Galindo A Schlembach D Zeisler H Herraiz I Moertl MG. An automated method for the determination of the sFlt-1/PIGF ratio in the assessment of preeclampsia. Am J Obstetr Gynecol. (2010) 202:161.e1–11. 10.1016/j.ajog.2009.09.016 19850276

[21] Pepe MS. The Statistical Evaluation of Medical Tests for Classification and Prediction. Oxford: Oxford University Press (2003).

[22] Zhang J Klebanoff MA Roberts JM. Prediction of adverse outcomes by common definitions of hypertension in pregnancy. Obstetr Gynecol. (2001) 97:261–7. 10.1097/00006250-200102000-00018 11165592

[23] Cerdeira AS O'Sullivan J Ohuma EO Harrington D Szafranski P Black R. Randomized interventional study on prediction of preeclampsia/eclampsia in women with suspected preeclampsia: INSPIRE. Hypertension. (2019) 74:983–90. 10.1161/HYPERTENSIONAHA.119.12739 31401877 PMC6756298

[24] Stepan H Hund M Dilba P Sillman J Schlembach D. Elecsys® and Kryptor immunoassays for the measurement of sFlt-1 and PlGF to aid preeclampsia diagnosis: are they comparable? Clin Chem Lab Med. (2019) 57:1339–48. 10.1515/cclm-2018-1228 31323000

[25] Lefevre G Hertig A Guibourdenche J Levy P Bailleul S Drouin D. Decision-making based on sFlt-1/PlGF ratios: are immunoassay results interchangeable for diagnosis or prognosis of preeclampsia? Clin Chem Lab Med. (2020) 59:e87–9. 10.1515/cclm-2020-0084 32238604

