# Supplementary material for: Short-Term Prediction of Preeclampsia in Chinese Women Using the Soluble fms-Like Tyrosine Kinase 1/Placental Growth Factor Ratio: A Sub-Analysis of the PROGNOSIS Asia Study
Source: Front Cardiovasc Med. 2021 Aug 23;8:602560. doi: 10.3389/fcvm.2021.602560 (PMC8419517; doi:10.3389/fcvm.2021.602560)
Supplement: Supplementary file 1 [file Data_Sheet_1.docx]

***Supplementary Material***

**Supplementary Figure 1:** Participant disposition.
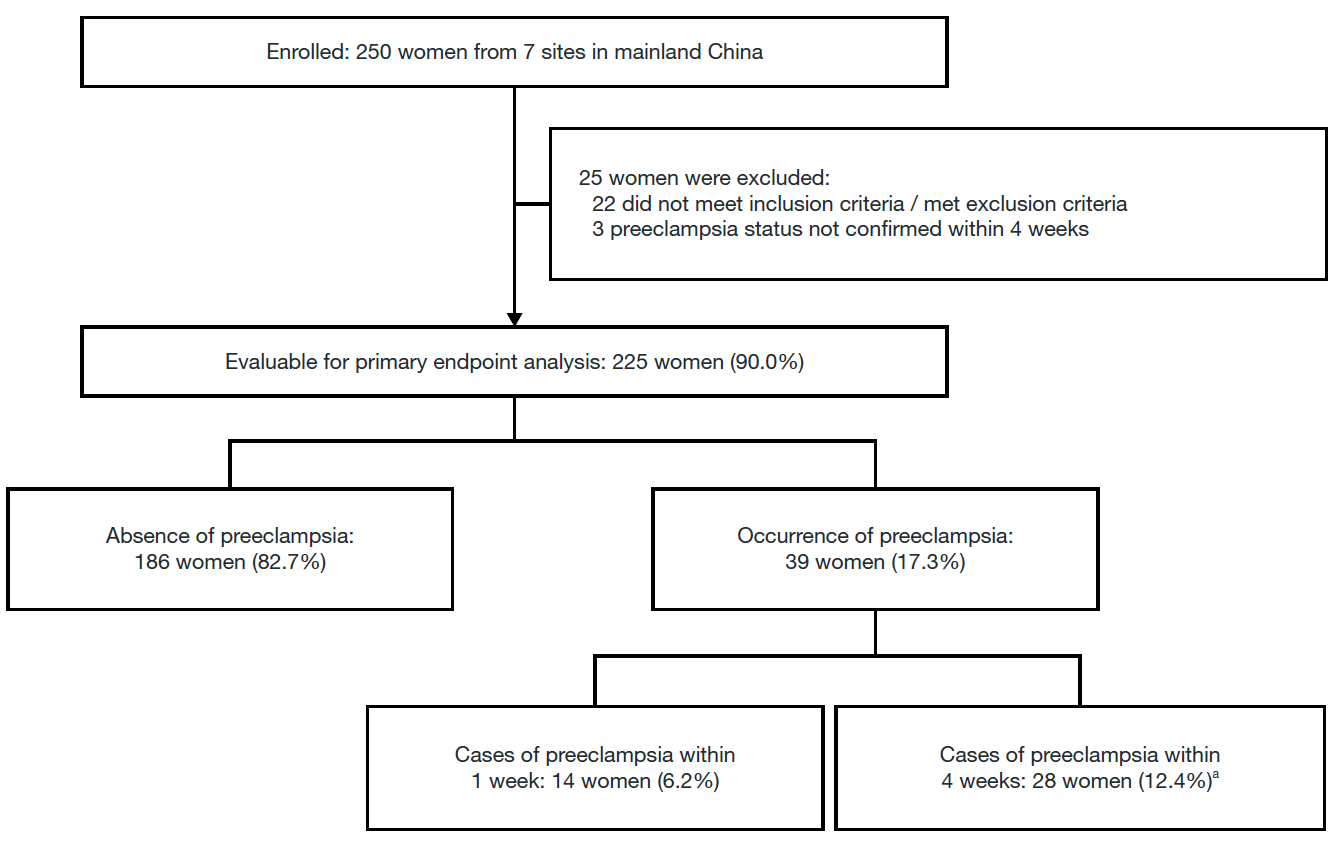


^a^Cases of preeclampsia occurrence within 4 weeks (*n* = 28) includes women for whom preeclampsia occurred within 1 week (*n* = 14).
